# Endoscopic Variceal Therapy With Multisystemic Glue Embolism: A Case Report and Narrative Review of a Rare but Severe Complication

**DOI:** 10.1155/crcc/4510213

**Published:** 2025-11-10

**Authors:** Monica Penati, Silvia Lazzaroni, Marine Neury, Karim Bendjelid

**Affiliations:** ^1^ Intensive Care Service, Department of Acute Medicine, Geneva University Hospitals, Geneva, Switzerland, hug-ge.ch; ^2^ Department of Clinical Pathology, Geneva University Hospitals, Geneva, Switzerland, hug-ge.ch

**Keywords:** cyanoacrylate glue embolization, decompensated cirrhosis, gastroesophageal variceal bleeding

## Abstract

Gastroesophageal variceal bleeding is a life‐threatening complication of portal hypertension in patients with cirrhosis. Endoscopic cyanoacrylate injection is an established therapy for variceal hemorrhage, but it may lead to rare systemic complications, including glue embolization. The authors report the case of a patient with decompensated cirrhosis who developed acute gastroesophageal variceal bleeding, managed with endoscopic variceal ligation and cyanoacrylate injection. Postprocedural investigations revealed multiple emboli involving the cerebral, pulmonary, coronary, and renal vascular territories. The present case report and review of the literature highlight the need for increased awareness of glue embolization as a potential adverse effect of cyanoacrylate therapy. Careful patient selection, refined endoscopic technique, and close postprocedure monitoring are essential to minimizing the risks of embolization, reducing complications and optimizing outcomes.

## 1. Introduction

Cirrhosis is a leading cause of global morbidity and mortality, frequently complicated by portal hypertension and its life‐threatening consequences, including gastroesophageal variceal bleeding [1, 2]. Nonselective beta‐blockers (NSBBs) and endoscopic band ligation (EBL) remain the cornerstones of primary and secondary prophylaxis of esophageal varices [3, 4]. However, advanced interventions including variceal cyanoacrylate injection, transjugular intrahepatic portosystemic shunt (TIPS), or self‐expanding metal stents (SEMS) may be required as second‐line treatments in selected patients and high‐risk cases [4, 5]. Among these treatments, cyanoacrylate injection is particularly effective for the management of variceal gastric bleeding, but it carries a risk of shunt with systemic embolism, a rare, but potentially fatal complication. This case report describes a unique instance of multiorgan glue embolisms following cyanoacrylate treatment, emphasizing the importance of early recognition, supportive care, and individualized risk‐benefit assessment in cirrhotic patients undergoing endoscopic treatment for variceal bleeding.

## 2. Case Presentation

A 45‐year‐old Caucasian female with a history of alcohol‐related Child C cirrhosis since 2021, alcohol dependence syndrome, and borderline personality disorder presented to the emergency department in December 2023 with melena over the past 4 days and a hematemesis episode the previous day. She also reported a weight gain of 3 kg over 3 days, accompanied by mild dyspnea and fatigue. Her medical history included a recent hospitalization (November 2023) for ascitic decompensation following inadequate diuretic therapy (spironolactone 50 mg once daily). Two Stage II esophageal varices were also treated with elastic band ligation as primary prophylaxis of variceal bleeding 2 weeks prior. Her current medications were spironolactone 50 mg twice daily, torasemide 2.5 mg once daily, bisoprolol 12.5 mg once daily, vitamin B1 100 mg once daily, and vitamin B12 one tablet once daily. Therapeutic adherence was good, but the patient had intermittent, unquantifiable alcohol consumption for the past 7 years.

On initial assessment, the patient was icteric and presented with lower limb edema. The abdomen was distended, with probable ascites. Rectal examination revealed melena. The patient was alert and responsive, with no signs of hepatic encephalopathy (HE). Her heart rate was 93 bpm. She had mild hypotension, with a blood pressure of 88/35 mmHg (mean arterial pressure of 53 mmHg), a temperature of 36°C, and an oxygen saturation of 96% on room air with a respiratory rate of 16 bpm.

Laboratory tests at admission indicated acute‐on‐chronic liver failure (ACLF) with multiorgan dysfunction, including hepatic failure (Factor V: 26%, normal 70%–120%), acute kidney injury classified as AKIN Stage I, and coagulopathy with prothrombin time of 24% (normal: > 70%), INR 2.61, activated partial thromboplastin time (aPTT) 54 s (normal: 26–37 s), and fibrinogen 1.8 g/L (normal: 2–4 g/L). At admission, the total bilirubin level was 218 *μ*mol/L (normal: 7–25 *μ*mol/L) with conjugated bilirubin of 75 *μ*mol/L (normal: 0.5–9.5 *μ*mol/L), alanine aminotransferase was 20 U/L (normal: 9–42 U/L), aspartate aminotransferase was 42 U/L (normal: 11–42 U/L), *γ*‐glutamyl‐transferase was 22 U/L (normal: 9–35 U/L), and alkaline phosphatase was 83 U/L (normal: 25–102 U/L). The blood count showed hemoglobin at 61 g/L (normal: 120–160 g/L), platelets at 46 G/L (normal: 150–450 G/L), and leukocytosis at 13.6 G/L (normal: 4–10 G/L) with elevated C‐reactive protein (CRP) at 15 mg/L (normal: < 5 mg/L).

On initial assessment, the patient presented with hemorrhagic shock prompting the administration of blood products (6 units of packed red blood cells, 4 units of plasma, and 6 g of fibrinogen) and norepinephrine. Terlipressin (2 mg/4 h) was also initiated. The patient is rapidly intubated in the emergency room for an urgent esophagoduodenoscopy due to suspected upper gastrointestinal bleeding. Empirical antibiotic therapy with intravenous ceftriaxone (2 g/day) was initiated alongside continuous intravenous high‐dose pantoprazole (8 mg/h).

Endoscopy revealed active spurting hemorrhage from a Grade III esophageal varix. Numerous large gastric varices without active bleeding were also identified. Multiple attempts at elastic variceal ligation (EVL) were unsuccessful. An emergency TIPS was placed between the right hepatic vein and right portal branch, reducing the portosystemic pressure gradient from 21 to 8 mmHg. Subsequently, under fluoroscopic guidance, four injections of cyanoacrylate glue (total dose 6 mL of Glubran, 1:2 dilution) were administered via the mesenteric venous circulation directly into the esophagogastric varices, with an effective obturation. Glue migration was observed in the inferior vena cava and pulmonary artery, likely due to high variceal flow. A second endoscopic attempt at variceal ligation was successful, achieving hemostasis without recurrence of bleeding.

During the endoscopic procedure, the patient′s hemodynamic status continued to deteriorate, requiring aggressive transfusion support (9 units of packed red blood cells, 3 units of platelets, and 6 units of plasma) and high‐dose norepinephrine, titrated up to 0.5 mcg/kg/h. Due to severe hemodynamic instability, the patient was rapidly admitted to the intensive care unit (ICU) without immediate further investigation of the glue migration.

She had hypotension despite aminergic support (blood pressure 78/34 mmHg, mean arterial pressure 50 mmHg), a temperature of 35.7°C. She was fully sedated and mechanically ventilated and required an FiO_2_ (fraction of inspired oxygen) of 55% to achieve 92% SpO_2_ (peripheral oxygen saturation). Terlipressin treatment was continued. Hydrocortisone (100 mg/8 h) was introduced along with methylene blue perfusion (100 mg/day for 3 days) to treat an associated vasodilatory shock, attributed to ischemia‐reperfusion following hemorrhagic shock and septic shock, due to possible aspiration pneumonia and bacterial translocation following variceal bleeding. Bedside abdominal ultrasound identified a small, undrainable amount of ascites, consistent with chronic liver disease and portal hypertension. Abdominal CT confirmed embolization of esophageal varices and the correct position of TIPS. The liver was dysmorphic with irregular contours and heterogeneous enhancement; a cystic lesion in Segment VI was present. The portal vein and the suprahepatic veins were patent, and the bile ducts were not dilated. Thoracic CT confirmed bilateral aspiration pneumonia. The recent hospitalization prompted a switch in antibiotic therapy to piperacillin/tazobactam (2.25/8 h). Bacteriological cultures (blood, urine, bronchoalveolar lavage, and pleural fluid), as well as *β*‐D‐glucan and blood galactomannan tests, were negative. No fever occurred during hospitalization. Coagulopathy‐related complications included a right spontaneous hemothorax, which was drained, and significant epistaxis, requiring cauterization.

The admission ECG revealed an inferior ST‐elevation myocardial infarction (STEMI) that was not present in the emergency room. Coronary angiography ruled out coronary arteriosclerotic disease but identified a thrombotic occlusion in a branch of the retroventricular artery. Due to ongoing hemorrhagic shock and severe coagulopathy, no therapeutic interventions were feasible.

The patient′s hemodynamic status gradually improved: Methylene blue was discontinued on Day 3 and norepinephrine on Day 5. Terlipressin was continued due to suspected hepatorenal syndrome. However, thrombocytopenia persisted at approximately 25 G/L and Factor V levels stabilized at 22%.

Following sedation interruption on Day 5, the patient remained in a coma with decorticate posturing and periodic breathing. A total body CT scan on Day 7 revealed multiple cerebral arterial occlusions caused by hyperdense material consistent with cyanoacrylate glue, along with widespread both supratentorial and infratentorial ischemic brain lesions (Figure 1a,b). Embolic lesions were also detected in the lungs and left kidney (Figure 1c,d). A right‐to‐left shunt was suspected, but no patent foramen ovale (PFO) was detected on bubble echocardiography, although a Valsalva maneuver could not be performed. The patient′s neurological status did not improve, and life support was withdrawn in agreement with the family. The patient died on Day 10.

Figure 1Total body CT scan with intravenous iodinated contrast. Cerebral CT scan findings showed multiple proximal and central cerebral arterial occlusions and subocclusions by hyperdense material compatible with cyanoacrylate glue, including the right Sylvian bifurcation, the left Sylvian artery at M2 and M3, and the tip of the basilar trunk extending to the posterior cerebral arteries. (a, b) Multiple corticosubcortical hypodense areas, both supratentorial and infratentorial including the left parieto–fronto–temporal region (left middle cerebral artery territory), left occipital lobe, right superior, and middle frontal regions, as well as smaller areas in the right parietal lobe and right cerebellum, are consistent with recent ischemic lesions. (c) Total body contrast‐enhanced CT revealed multiple spontaneously hyperdense foci in the left bronchial vein and in the veins of both lower lobes. (d) Multiple cortical‐medullary infarcts were identified in the left kidney, while other abdominal organs showed no focal lesions. These findings support the diagnosis of systemic glue embolism following prior variceal embolization.

**Figure:**
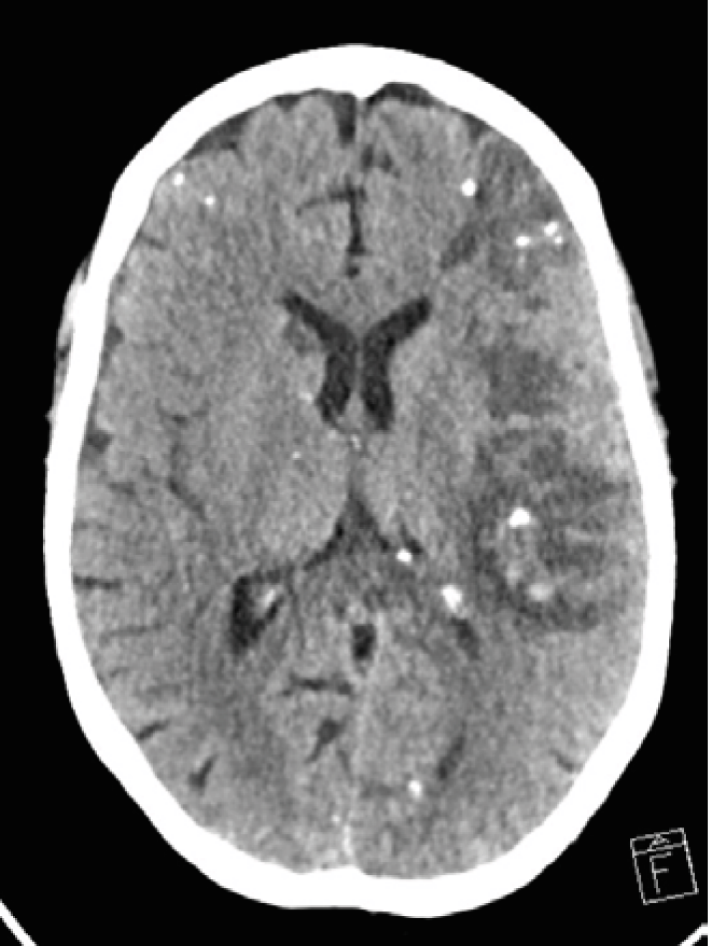
(a) Brain, axial plane

**Figure:**
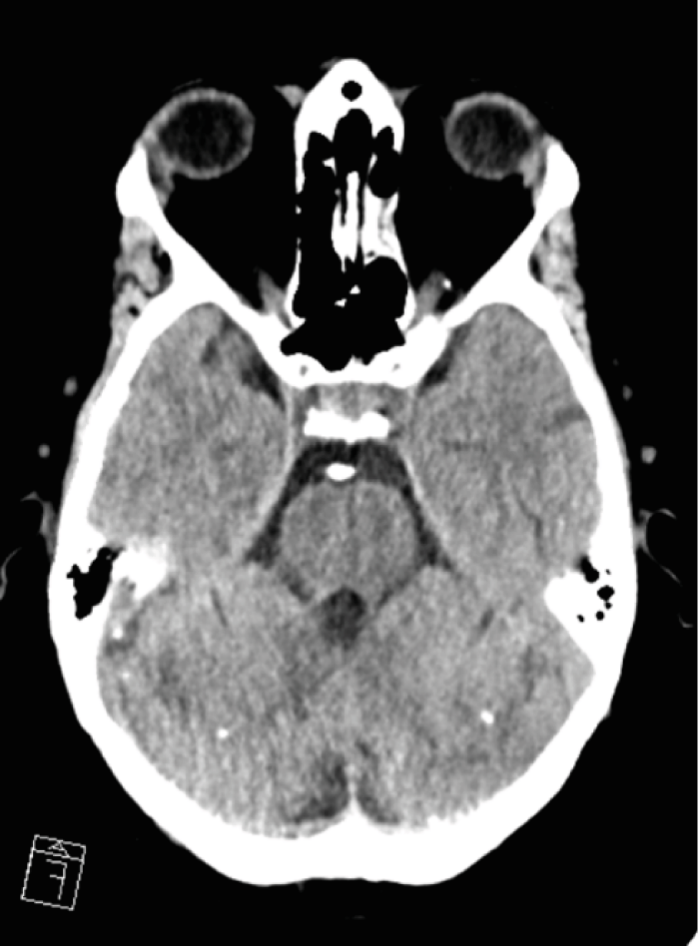
(b) Brain, axial plane

**Figure:**
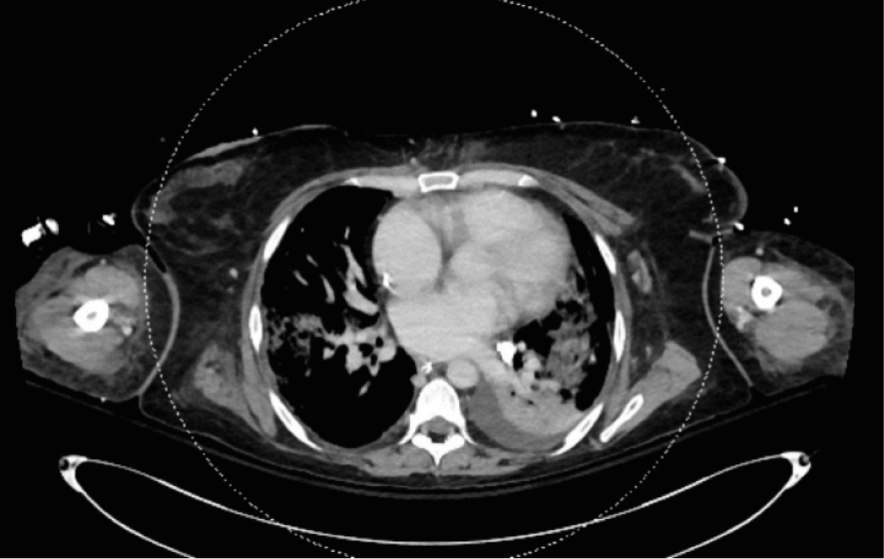
(c) Thorax, axial plane

**Figure:**
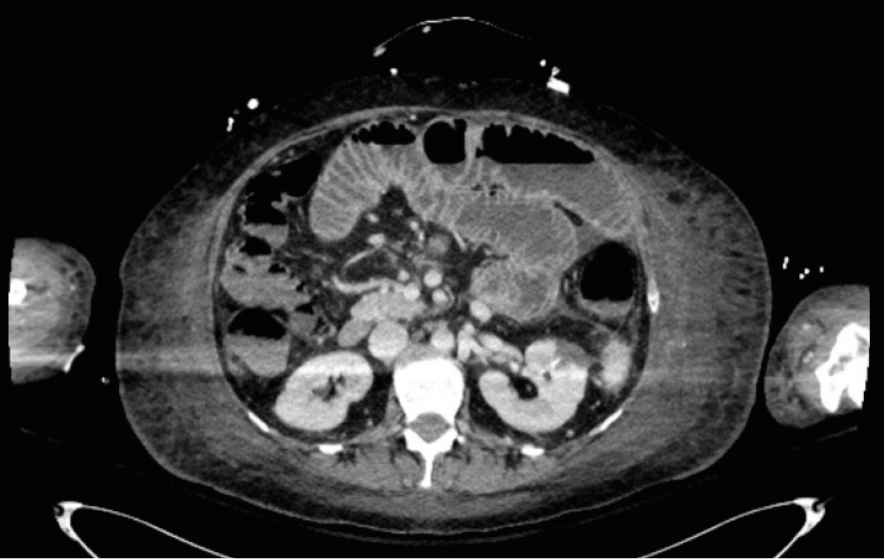
(d) Abdomen, axial plane

An autopsy was performed. At macroscopic examination, a dysmorphic micronodular liver reflected advanced cirrhosis, with signs of portal hypertension consisting of splenomegaly and gastroesophageal varices. The TIPS was correctly positioned and patent. One large gastroesophageal serosal vein (Figure 2a) was filled with embolization material (Figure 2b). Esophageal mucosa showed small erosions, without evidence of recent active bleeding.

Figure 2Macroscopic and microscopic autoptic findings. (a, b) An esophagogastric large varicose vein (0.8 cm, arrows) on the serosal surface was filled with a reddish, granular material, histologically translated as an intraluminal amorphous eosinophilic material mixed with blood and fibrin, consistent with embolization material. (c) A large pulmonary artery filled with a reddish granular material (arrow). (d) The microscopic aspect of the latter is identical to the material in the esophagogastric varix. (e) Multiple cortical infarctions of the left kidney. (f) A brownish epicardial discoloration (circle) of the posterior wall suggestive of ischemic infarction. (g) Partly hemorrhagic parenchymal brain lesions involving the left parietal lobe and extending to the insula, corresponding with acute infarction. (h) Similar lesions were objectified in both cerebellar hemispheres. The brainstem was unaffected.

**Figure:**
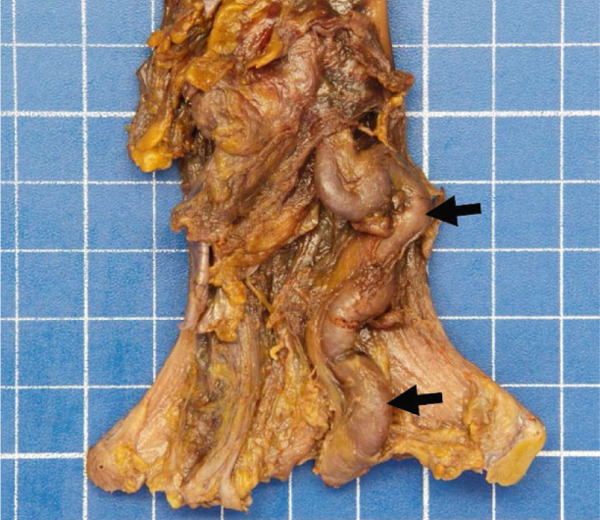
(a) Esophagogastric junction, external view

**Figure:**
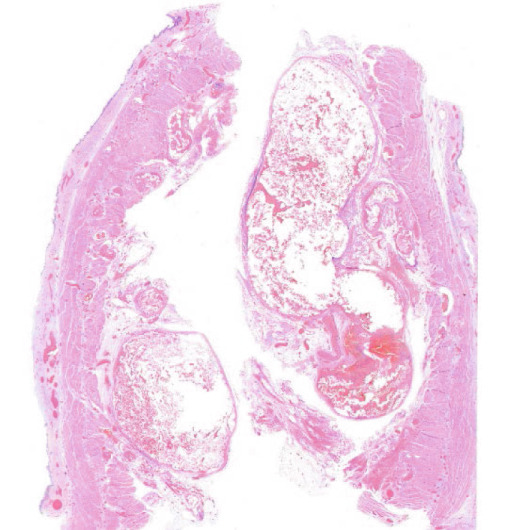
(b) Esophagogastric vein with intraluminal material

**Figure:**
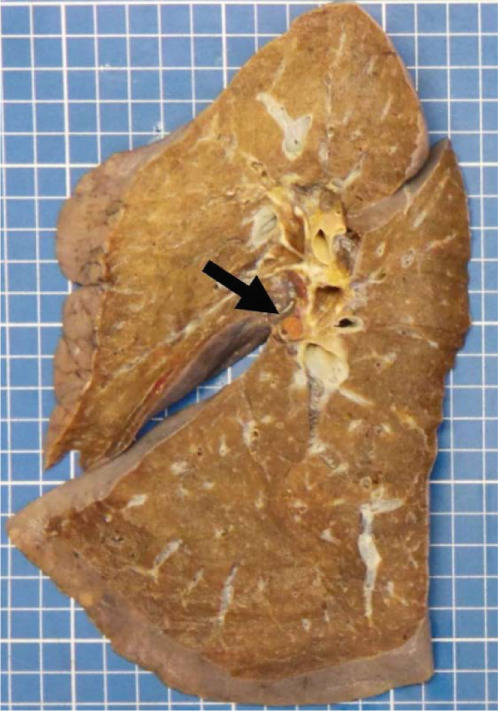
(c) Left lung, sagittal section

**Figure:**
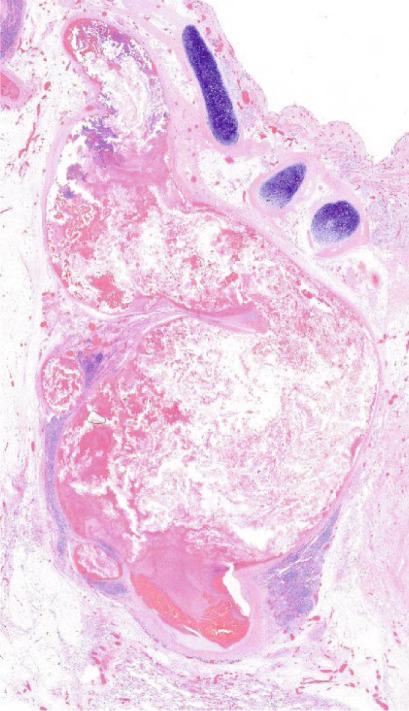
(d) Left lung, intravascular embolization material

**Figure:**
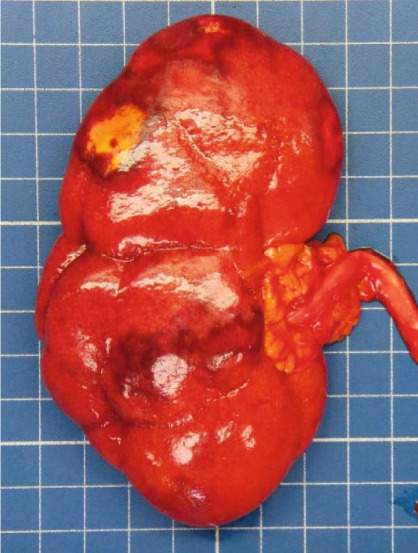
(e) Left kidney

**Figure:**
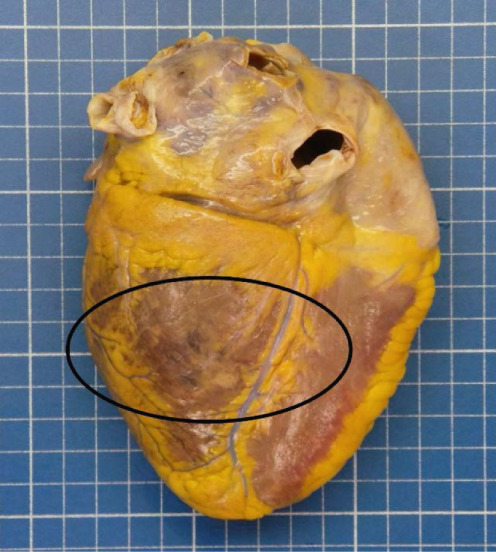
(f) Heart, posterior view

**Figure:**
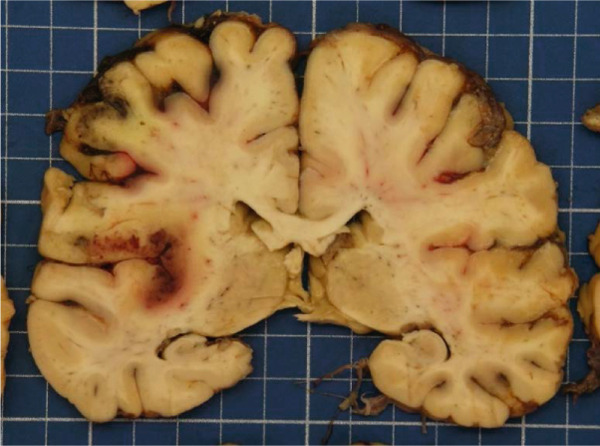
(g) Brain hemispheres, coronal section

**Figure:**
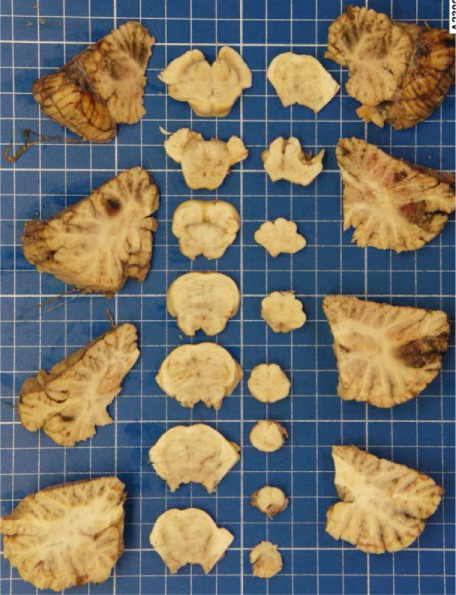
(h) Cerebellum and brainstem

A granular material was found in a large postcentral artery of the left lung (Figure 2c,d), with a gross and histological appearance identical to that found in the gastroesophageal vein. In the right lung, an extensive hemorrhagic parenchymal lesion of the inferior lobe was visualized, histologically consistent with an acute infarction with a hemorrhagic component. No evident embolization material was detected in the vascular spaces of the right lung, either on gross examination or on histological analysis.

Parenchymal lesions corresponding to acute infarctions were also observed in the left kidney (Figure 2e), myocardium (Figure 2f), and brain, with left‐predominant hemispheric damage (Figure 2g) as well as bilateral cerebellar involvement, with sparing of the brainstem (Figure 2h). Despite meticulous examination and extensive sampling, embolization material was not identified in those locations, nor was arteriosclerosis objectified in the coronary arteries.

These multiple infarctions in different locations may have occurred as part of an embolic event. However, the etiology explaining a systemic involvement or shunt between the venous and arterial circulations remained unclear at autopsy. Notably, although a discrete cribriform aspect of the interatrial wall was noted, no evidence of a PFO could be proven with a probe examination.

## 3. Discussion

Cirrhosis is a major cause of morbidity and mortality in patients with chronic liver failure, accounting for 2.4% of global death [1]. Its epidemiology varies by geographic and social context, but the most common causes of cirrhosis include alcohol abuse, hepatitis B and C, nonalcoholic fatty liver disease, and their overlap [1].

Cirrhosis, caused by chronic liver injury, leads to structural changes in the liver with the disruption of hepatic architecture, an imbalance of vasoactive substances and chronic inflammation, increasing intrahepatic resistance and resulting in portal hypertension [6]. When the hepatic venous pressure gradient (HVPG, calculated as the difference between wedged and free hepatic venous pressure, normally lower than 5 mmHg) exceeds 10 mmHg, complications such as portosystemic shunting, systemic vasodilatation, ascites, and esophageal and gastric varices may occur [3]. Portal hypertension is a key driver of severe cirrhosis‐related complications, including ascites, HE, and variceal bleeding [6, 7]. Despite their clinical impact, varices are not included in standard prognostic scores like Child–Pugh and MELD, even though variceal bleeding is a major cause of morbidity and mortality in cirrhotic patients [8].

The exact risk factors and triggers of variceal bleeding remain unclear, but key contributors include variceal pressure, variceal size, severity of liver disease, and the use of prophylactic treatment [9]. Portal pressure reflects variceal pressure: An increase in the HVPG > 10 mmHg is required for the development of gastroesophageal varices, while an HVPG > 12 mmHg is necessary for variceal bleeding. However, the relationship between portal hypertension severity and variceal hemorrhage risk is not strictly linear [9]. Endoscopy is the most reliable method to assess varices. Small varices collapse easily, while larger ones are more resistant and carry a higher risk of bleeding, especially if red markings are present. Bleeding risk increases with variceal size, liver disease severity (Child–Pugh score), and cirrhosis decompensation [9]. Patients with compensated cirrhosis and small varices have a 6% 1‐year bleeding risk, while those with large varices and decompensated cirrhosis may face up to a 76% risk [10]. In‐hospital mortality after variceal bleeding is around 14.5% [11].

NSBBs (propranolol, carvedilol, or nadolol) reduce the 3‐year risk of cirrhotic decompensation and mortality in patients with compensated advanced chronic liver disease and clinically significant portal hypertension [3, 8, 12]. Carvedilol is preferred for preventing variceal bleeding due to its superior ability to lower portal pressure, thanks to its alpha‐blocking effects [3, 4]. It is more effective than band ligation in high‐risk patients (large varices, red signs, and Child–Pugh C) [3, 9]. When NSBBs are contraindicated, EBL of high‐risk esophageal varices is recommended to prevent initial bleeding, with sessions repeated every 2–4 weeks until variceal eradication [3, 4].

Variceal bleeding is a medical emergency requiring a multidisciplinary approach. Treatment focuses on stabilizing the patient by managing hemorrhagic shock, restoring perfusion, and administering blood and coagulation support. Reducing portal hypertension is the main resuscitation goal rather than correcting coagulation abnormalities. Vasoactive drugs like vasopressin and somatostatin, along with their synthetic analogs terlipressin and octreotide, help control bleeding and should be started immediately, continuing for 2–5 days [3, 13].

Antibiotic prophylaxis is recommended before and shortly after endoscopy to reduce infection rate and rebleeding risk, especially targeting gram‐negative enteric bacteria. Though the link between bleeding and infection is unclear, invasive procedures (such as endoscopy, TIPS placement, variceal embolization, or ascites drainage) may compromise mucosal barriers and facilitate infection. Hematemesis, endoscopic interventions, and balloon tamponade increase the risk of aspiration pneumonia. In cirrhotic patients, factors such as bacterial translocation, complement deficiency, and hypovolemia further predispose them to infection [14]. Local antimicrobial policies, resistance patterns, and patients′ allergies should guide the choice of antibiotic prophylaxis [15].

HE occurs in up to 40% of cirrhotic patients with upper gastrointestinal bleeding and is associated with increased mortality. This is primarily due to hyperammonemia, resulting from the digestion of blood proteins, compounded by liver dysfunction, systemic inflammation, and infection. Lactulose therapy has been shown to improve survival and is recommended in cases of bleeding with concomitant HE [4].

Proton pump inhibitors (PPIs) are often administered before endoscopic examination, but they should be stopped once variceal bleeding is confirmed, as their use in cirrhotic patients increases the risk of bacterial infections, particularly spontaneous bacterial peritonitis and infections caused by multidrug‐resistant organisms, unless clearly indicated [4]. Endoscopy should be performed within 12 h after stabilization, or sooner if unstable [16, 17]. Intubation is advised for altered consciousness or vomiting. Pre‐endoscopic administration of erythromycin should be considered before endoscopy to improve visualization. Abdominal imaging is useful to rule out splanchnic vein thrombosis or liver cancer. Endoscopic diagnosis is based on the visualization of active bleeding from a varix or on the presence of signs of recent bleeding (red spots, nipple signs, and fibrin plug).

In case of acute esophageal variceal hemorrhage, EBL is the recommended method to control bleeding [4, 18]. The bands are applied on esophageal varices starting at the site of active or recent bleeding, if visible. The remaining varices are then treated beginning from the gastroesophageal junction and continuing in a spiral cephalad manner. The number of ligations impacts the procedure time and is associated with an increased risk of rebleeding [4].

Acute gastric variceal bleeding is usually more severe than esophageal hemorrhage, with higher associated mortality and treatment failure [19]. The use of cyanoacrylate injections is usually reserved for bleeding of isolated gastric varices (Type I) and gastroesophageal varices (Type II) for faster and more effective control of bleeding [3]. It is also used as a second‐line treatment for esophageal varices where banding is not possible due to degraded esophageal tissue or previous failed banding attempts [20]. Despite the very high technical success rate of 94.1% for cyanoacrylate embolization in the treatment of gastrointestinal bleeding, there is a moderate 30‐day rebleeding rate of 24.2% and a 5.3% risk of 30‐day major complications [21].

The trade name of cyanoacrylate glue varies depending on the country, but its chemical composition remains unchanged. It consists of a monomer of n‐butyl‐2‐cyanoacrylate, which undergoes polymerization upon contact with ionic substances, such as water or blood, forming a solid mass. This process leads to the occlusion of the vascular lumen and promotes local thrombosis. Cyanoacrylate glue is commonly mixed with Lipiodol (ethiodized oil) which slows the polymerization process, allowing more time for injection. Being radio‐opaque, it also permits postprocedural radiological examination. The tendency of the glue in its liquid phase to spread along vessels both distal and proximal to the bleeding varix should be emphasized, as it plays a crucial role in local venous thrombosis and distant embolization. Additionally, specific hemodynamic characteristics of cirrhotic patients such as hyperdynamic circulation, the presence of artificial portosystemic shunts following the placement of a TIPS, and dilated pulmonary vessels (hepatopulmonary syndrome) can facilitate its migration within the vasculature [20].

Embolization is the main complication of cyanoacrylate injections (reported incidence: 0.5%–4.3%) [22]. The glue injected into the branches of the portal system migrates following gradient pressure into the inferior cava vein via the TIPS. Further migration and embolization to the right atrium or pulmonary artery are well known and can present with highly variable clinical features, ranging from asymptomatic cases to symptoms such as dyspnea, pleuritic chest pain, cough, tachycardia, hypoxia, and, in severe cases, cardiorespiratory arrest or sudden death [23]. The onset of respiratory symptoms varies widely, occurring anywhere from a few minutes to several hours after cyanoacrylate injection [24]. It is important to note that imaging findings do not correlate well with the clinical condition, as radiographic features of glue emboli may persist despite the patient′s clinical improvement. The management of patients with pulmonary glue emboli is mainly supportive, and there is usually no need for thrombolysis or anticoagulation [25]. In the case of intra‐ or extracardiac left‐to‐right shunt, systemic embolization of the glue into the arterial system is possible. Cerebral embolization has been reported multiple times, and it is a potentially life‐threatening complication [26, 27]. It should be suspected in patients exhibiting abnormal neurological recovery following variceal embolization. Even in this circumstance, management remains supportive. Investigations for a PFO are recommended. Coronary glue embolization is a rare complication with an insidious clinical presentation, ranging from asymptomatic cases to symptoms such as arrhythmias, acute coronary syndrome, and cardiorespiratory arrest or sudden death [28]. There is limited evidence in the literature regarding the treatment of coronary glue embolization. However, given its known thrombogenic potential, anticoagulation, adjusted to the patient′s bleeding risk, is likely necessary [29]. In general, any organ can be affected by glue embolization. A high index of clinical suspicion is essential when assessing patients for possible procedure‐related complications. In the presented case, both pulmonary (pulmonary artery) and systemic (cerebral, coronary, and renal involvement) embolization are evident. The exact mechanism of systemic arterial embolization in our patient remains unclear; however, potential pathways include passage of glue through a PFO or via a cirrhosis‐induced pulmonary shunt (hepatopulmonary syndrome).

Glue injection for varices can lead to local venous thrombosis, particularly in the portomesenteric vessels due to their proximity. Although its impact on morbidity and mortality remains unknown, clinical experience suggests that new portal vein thrombosis is likely detrimental to patients with cirrhosis [20]. Much rarer complications are fistulization due to extravascular injection, ulceration and erosion of the variceal wall, variceal laceration, septic thrombophlebitis, hypersensitivity, and allergic reactions [20].

Postbleeding management depends on liver disease severity. A HVPG ≥ 20 mmHg best predicts worse outcomes, but MELD and Child–Pugh scores are commonly used for risk stratification [30–32].

Patients with Child–Pugh A or B (without active bleeding) or MELD < 11 should be considered at lower risk of poor outcome. After successful band ligation, vasoactive drugs should be continued for up to 5 days. NSBBs should be started or resumed, and a follow‐up endoscopy within 1–4 weeks is recommended to eradicate esophageal varices as a secondary prophylaxis [4].

Patients presenting with a MELD score ≥ 19, Child–Pugh B with active bleeding, or a Child–Pugh C are at high risk of poor outcome. High‐risk patients (those with HVPG ≥ 20 mmHg at the time of bleeding, Child–Pugh C ≤ 13, and Child–Pugh B > 7 with active bleeding despite vasoactive agents) should undergo preemptive TIPS within 72 h (ideally within 24 h) after successful variceal bleeding control [31–33]. TIPS, performed by an interventional radiologist, reduces portal pressure by creating a shunt between the portal and hepatic veins. Achieving an HVPG < 12 mmHg is the goal for hemodynamic success [3]. Preemptive TIPS lowers rebleeding and improves ascites control without increasing encephalopathy risk and may reduce mortality in high‐risk groups compared to standard of care (vasoactive agents and endoscopy) [31, 34, 35]. The survival benefit of preemptive TIPS remains controversial in lower risk patients (Child–Pugh B) and in those at a higher risk (Child–Pugh C ≥ 14 points, MELD > 30, or lactates > 12 mmol/L) for whom TIPS could be futile [4, 36]. Nonetheless, ACLF, HE, and hyperbilirubinemia are not contraindications to TIPS [4, 31].

TIPS is an established salvage treatment in cases where esophageal variceal bleeding persists despite adequate vasoactive and endoscopic therapy. When TIPS is not immediately feasible or in refractory EVH, balloon tamponade (e.g., Sengstaken–Blakemore or Minnesota tube) can control bleeding temporarily but carries risks like esophageal ulceration or perforation and aspiration pneumonia [4]. SEMS offer a safer alternative, can stay in place for up to 14 days, and are effective for tamponade. Both devices can be used as a bridge to definitive hemostasis or rescue TIPS [5, 37].

Early rebleeding within 5 days occurs in up to 20% of patients, especially those with hepatocellular carcinoma, advanced liver disease, or low beta‐blocker use. Causes include band slippage, postbanding ulcers, and esophageal necrosis. A second attempt at endoscopic treatment and/or salvage TIPS should be performed in these cases [4, 38].

## 4. Conclusion

Cyanoacrylate injection is an effective therapeutic option for controlling bleeding from gastric varices and serves as a second‐line treatment for esophageal variceal hemorrhage. However, clinicians should remain vigilant for rare but potentially life‐threatening complications such as systemic glue embolization. This case highlights the importance of early recognition, supportive management, and individualized risk assessment in patients undergoing endoscopic therapy. A multidisciplinary approach, careful procedural planning, and monitoring are essential for reducing complications and improving patient outcomes.

## Ethics Statement

No ethical approval was required due to the nature of the manuscript.

## Consent

Written informed consent was obtained from the patient′s family for publication of this case report and accompanying images. A copy of the written consent is available for review by the editor‐in‐chief of this journal on request.

## Disclosure

All authors reviewed and approved the final manuscript.

## Conflicts of Interest

The authors declare no conflicts of interest.

## Author Contributions

**Monica Penati:** writing – review and editing, writing – original draft, conceptualization. **Silvia Lazzaroni:** writing – review and editing, writing – original draft, conceptualization. **Marine Neury:** provided macro‐ and histopathological analysis, writing – review and editing. **Karim Bendjelid:** writing – review and editing. Monica Penati and Silvia Lazzaroni contributed equally to the original manuscript and to the redaction of this paper.

## Funding

No funding was received for this manuscript.

## Data Availability

Data sharing not applicable to this article as no datasets were generated or analysed during the current study.
